# Investigation of Patient-Specific Maxillofacial Implant Prototype Development by Metal Fused Filament Fabrication (MF^3^) of Ti-6Al-4V

**DOI:** 10.3390/dj9100109

**Published:** 2021-09-23

**Authors:** Mohammad Qasim Shaikh, Subrata Deb Nath, Arulselvan Arumugam Akilan, Saleh Khanjar, Vamsi Krishna Balla, Gerald Thomas Grant, Sundar Vedanarayanan Atre

**Affiliations:** 1 Materials Innovation Guild, University of Louisville, Louisville, KY 40208, USA; mohammadqasim.shaikh@louisville.edu (M.Q.S.); subrata.nath@louisville.edu (S.D.N.); arulselvan.arumughamakilan@louisville.edu (A.A.A.); saleh.khanjar@louisville.edu (S.K.); vamsi.balla@gmail.com (V.K.B.); 2 Bioceramics and Coating Division, CSIR-Central Glass and Ceramic Research Institute, 196 Raja S.C. Mullick Road, Kolkata 700 032, India; 3 School of Dentistry, University of Louisville, Louisville, KY 40202, USA; gerald.grant@louisville.edu

**Keywords:** maxillofacial restoration, patient-specific implant, metal fused filament fabrication, additive manufacturing, biomedical, simulation, titanium Ti-6Al-4V

## Abstract

Additive manufacturing (AM) and related digital technologies have enabled several advanced solutions in medicine and dentistry, in particular, the design and fabrication of patient-specific implants. In this study, the feasibility of metal fused filament fabrication (MF^3^) to manufacture patient-specific maxillofacial implants is investigated. Here, the design and fabrication of a maxillofacial implant prototype in Ti-6Al-4V using MF^3^ is reported for the first time. The cone-beam computed tomography (CBCT) image data of the patient’s oral anatomy was digitally processed to design a 3D CAD model of the hard tissue and fabricate a physical model by stereolithography (SLA). Using the digital and physical models, bone loss condition was analyzed, and a maxillofacial implant initial design was identified. Three-dimensional (3D) CAD models of the implant prototypes were designed that match the patient’s anatomy and dental implant requirement. In this preliminary stage, the CAD models of the prototypes were designed in a simplified form. MF^3^ printing of the prototypes was simulated to investigate potential deformation and residual stresses. The patient-specific implant prototypes were fabricated by MF^3^ printing followed by debinding and sintering using a support structure for the first time. MF^3^ printed green part dimensions fairly matched with simulation prediction. Sintered parts were characterized for surface integrity after cutting the support structures off. An overall 18 ± 2% shrinkage was observed in the sintered parts relative to the green parts. A relative density of 81 ± 4% indicated 19% total porosity including 11% open interconnected porosity in the sintered parts, which would favor bone healing and high osteointegration in the metallic implants. The surface roughness of Ra: 18 ± 5 µm and a Rockwell hardness of 6.5 ± 0.8 HRC were observed. The outcome of the work can be leveraged to further investigate the potential of MF^3^ to manufacture patient-specific custom implants out of Ti-6Al-4V.

## 1. Introduction

For the success of dental implants, anatomic conditions such as sufficient bone height, thickness, and density, play a deciding role [1,2,3]. Bone regeneration by grafting is widely employed to grow new bone in weak jawbone areas by autografting, using other bone as a scaffold [4,5]. However, in case of severe bone resorption, extensive bone regeneration requirement represents clinical treatment challenges leading to hesitation from patients [6]. The development of a patient-specific implant would suffice the need for adequate bone structure to support dental implants. Particularly for elderly patients, such an implant is of great importance as they cannot or may not want to undergo complex regenerative surgeries, but need a fixed dental restoration [7,8]. Apart from dental rehabilitation, maxilla and mandible reconstructions find applications in treating bone defects caused by tumors, injuries, or infections [9,10]. However, such reconstruction represents major challenges from both engineering and medical aspect [9]. Firstly, the complexity of facial anatomy, vital adjacent organs, the possibility of infection, and the uniqueness of each patient are the challenge for doctors. Secondly, complex facial bone structure design, the unique morphology of each patient, high demand on reconstruction material and performance, and limitations of the manufacturing process pose great deal challenges for engineers [11]. Thirdly, high osteoporotic structure in elderly patients makes it more challenging for doctors due to low regeneration tendency and overall capability to sustain, and for engineers due to reduced bone structure area and strength to support custom implants. However, several developments in digital technology have made it feasible to fabricate custom-made implants that perfectly match the anatomy and local morphology of the patient [7,12]. Modern technologies such as cone beam computed tomography (CBCT) for patient data acquisition, high-speed intraoral scanner to capture a direct optical impression, digital software for clinical analyses and surgical planning, 3D printers for a wide range of high-performance materials have fueled the progress in implant dentistry and maxillofacial reconstruction [8,11,13].

Additive manufacturing (AM) has enabled the fabrication of patient-specific implants for individual patients [9,14,15,16]. Moreover, the AM process can produce porous structures that help in optimizing the effective stiffness, and thus reduce stress shielding in implants [13]. Such porosity also provides anchor sites to the bone tissue and promotes accelerated osseointegration [3]. Hence, AM implants could adequately transfer the stresses between implant and bone, thereby increasing the life of the implant and implant-supported restoration. While AM brings in promising capabilities for dental and maxillofacial implants, from the material front, limited compatible choices are available due to versatile demands on mechanical, physical, and chemical characteristics of the implant material [17,18]. Among others, titanium is a widely used material in implants and other biomedical applications due to its high strength-to-weight ratio, corrosion resistance, low density, and non-magnetic properties [13,19]. Due to its high biocompatibility, Ti-6Al-4V is considered one of the most suitable biomaterials for medical applications [20]. Fabrication of Ti-6Al-4V has been investigated with multiple AM technologies [21].

Metal AM technologies such as selective laser melting (SLM), electron beam melting (EBM) or direct energy deposition (DED) have been widely explored for metal implant fabrications [19,22,23]. However, the limitations of these processes are very high initial capital investment and safety concerns due to direct working with loose reactive metal powder. Moreover, the high energy consumption of the only choice of industrial level operation limits the economic viability of small-batch manufacturing. In addition, high thermal gradients, localized heat, and rapid cooling rates induce residual stresses, distortion, non-equilibrium microstructures, and anisotropy leading to structural property differences [24,25]. Moreover, the quality of the product is a major challenge facing any AM technology. High dimensional accuracy, optimal microstructural and mechanical properties with required repeatability and reproducibility continue to remain the need, and hence, the focus for AM research in biomedical implants development [26]. In addition, due to the small batch size or highly customized production, traditional methods of quality assurance may not be appropriate for AM products. Furthermore, very limited information is available on AM-printed Ti-6Al-4V part evaluation as it goes through various stages of manufacturing. These limitations act as a barrier to the widespread implementation of metal AM technologies in implant dentistry and maxillofacial reconstructions. To overcome the above limitations, an advanced AM technology, known as metal fused filament fabrication (MF^3^), is rapidly emerging. It enables the fabrication of metal parts using desktop-level FFF printers [16,27,28,29,30,31,32].

**Figure 1:**
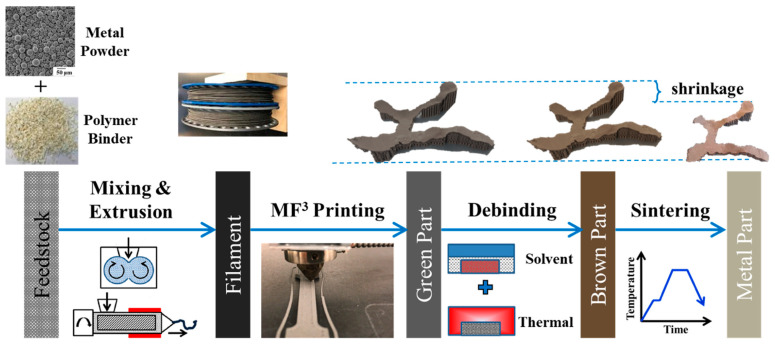
Overview of MF^3^ process showing filament preparation, 3D printing, debinding and sintering, and demonstration of an implant prototype fabricated by MF^3^ with Ti-6Al-4V alloy.

MF^3^ is an extrusion-based printing process that uses highly filled metal powder-polymer binder filaments, where the polymer binder helps to hold metal particles together in a feedstock and assists in material flow and deposition during printing. As shown in Figure 1, the MF^3^ process starts with sinterable metal powder, which is Ti-6Al-4V in this study, bonded in a multi-component polymer-based binder [27,28,29,30,31,32]. The metal powder content generally varies between 55 to 60% volume of the powder-binder mixture. The feedstock is extruded to form a 1.75 mm diameter filament that can be used on an extrusion-based desktop printer to build a 3D part. The printed part referred to as the ‘green part’, is subsequently subjected to debinding to remove polymer binder leading to a ‘brown part’. Finally, sintering is conducted in an inert environment using H_2_ or N_2_ gas at elevated temperatures. This completes the cycle providing a fully dense metal part.

MF^3^ has been successfully used to print Ti-6Al-4V parts of varying geometries, as reported in the previous publications by our research group [27,28,30,31,32]. MF^3^ sintered Ti-6Al-4V parts exhibited an ultimate tensile strength of 875 ± 15 MPa and 17 ± 3% elongation at 94.1 ± 0.1% relative density which are comparable to metal injection molded (MIM) parts [29]. The higher the relative density, the lower the porosity/voids and the greater the mechanical properties. The microstructures in different directions of the sintered parts were found to be isotropic throughout and are comparable to MIM microstructures. It was identified in the literature survey that implant dentistry and maxillofacial reconstruction have a pressing need for fabrication technologies that could manufacture custom-made implants efficiently and economically at small to moderate scales. Building on the findings in Ti-6Al-4V printing with MF^3^, in this work, we investigated the feasibility and suitability of Ti-6Al-4V printing with MF^3^ to manufacture patient-specific maxillofacial implant prototypes.

The purpose of this investigation is to examine the applicability of MF^3^ to manufacture a patient-specific maxillofacial implant for dental restoration of elderly patients with osteoporotic maxillary structure. We described the methodology followed in the design and fabrication of Ti-6Al-4V maxillofacial implants using MF^3^ technology. The methodology from processing the digital data of the patient’s oral anatomy to design development and fabrication of implant prototypes is discussed. There was a specific emphasis on the applicability of MF^3^ for maxillofacial implant geometries in terms of manufacturability with the inclusion of support structures for the first time. Furthermore, MF^3^ printing of the implants was simulated to investigate potential deformation and residual stresses. Moreover, the sintered parts were characterized for surface integrity in terms of surface topography, density, porosity, microstructure, and hardness that would affect the implant performance.

This study is based on a real clinical case of an 85-year-old partially edentulous female patient. With the complaints of difficulty in eating and speech, she intended to get dental restoration. Her CBCT scan revealed severe resorption of the upper jaw and maxillary bone and no teeth in the upper jaw. To provide for dental implants, adequate reconstruction of the maxillary structure was needed. Considering the patient’s age, bone regeneration was not a suitable option. Hence, the custom-made maxillofacial implant was the best solution.

## 2. Materials and Methods

The workflow started with the patient’s anatomical data in 2D DICOM format obtained from CBCT scan. This data was imported into a biomedical software, Mimics (Materialise, Leuven, Belgium), for image processing and segmentation to develop a 3D CAD of the patient’s facial bone and dental structure. This 3D model in STL format was used to fabricate a physical biomodel by SLA process. A photocurable acrylate material FLGPWH04 (Formlabs, Somerville, MA, USA) was used to fabricate the anatomical model by the SLA method using a Form-2 (Formlabs, Somerville, MA, USA) printer. The biomodel helped the oral and maxillofacial surgeons to evaluate the current condition of the maxilla structure and implant requirements and accordingly propose a patient-specific implant solution. Using this input, an initial design of the implant was developed matching the patient’s maxilla structure, and 3D CAD of the implant prototypes was generated using modeling software, 3-Matic (Materialise, Leuven, Belgium), considering the maxilla structure geometry as reference. Using the STL files, implant components were printed by MF^3^ with the filament which has 59% volume of Ti-6Al-4V powder dispersed in a multi-component custom polymer matrix using a desktop printer, Pulse (MatterHackers, Lake Forest, CA, USA). The green parts were then debound and sintered to get fully dense Ti-6Al-4V parts. Apart from physical printing, the MF^3^ printing process was also simulated using a CAE simulation tool, Digimat (MSC Software, Newport Beach, CA, USA), to estimate part deflections and residual stresses. Finally, the resulting parts were characterized for surface integrity attributes, such as geometric fidelity, density, porosity, surface morphology, metallography, and hardness were evaluated. A typical workflow of patient-specific implant fabrication using the MF^3^ process is presented as the one followed for the case study analyzed.

### 2.1. Design of the Implant

#### 2.1.1. Image Processing and Segmentation

The latest facial morphology of the patient was obtained through a CBCT scan in 2D DICOM format. This 2D data was imported into Mimics for image processing and segmentation, and a 3D model of facial anatomy was generated from the 2D images. The vital aspect of this process was extracting the region of interest from DICOM images without much compromise to actual anatomical details. The Mimics tool provides a feature called segmentation to separate the anatomical regions (hard and soft regions) using the Hounsfield radiodensity scale. The hard bone elements were segmented by filtering out a radiodensity of less than ~610 HU.

**Figure 2:**
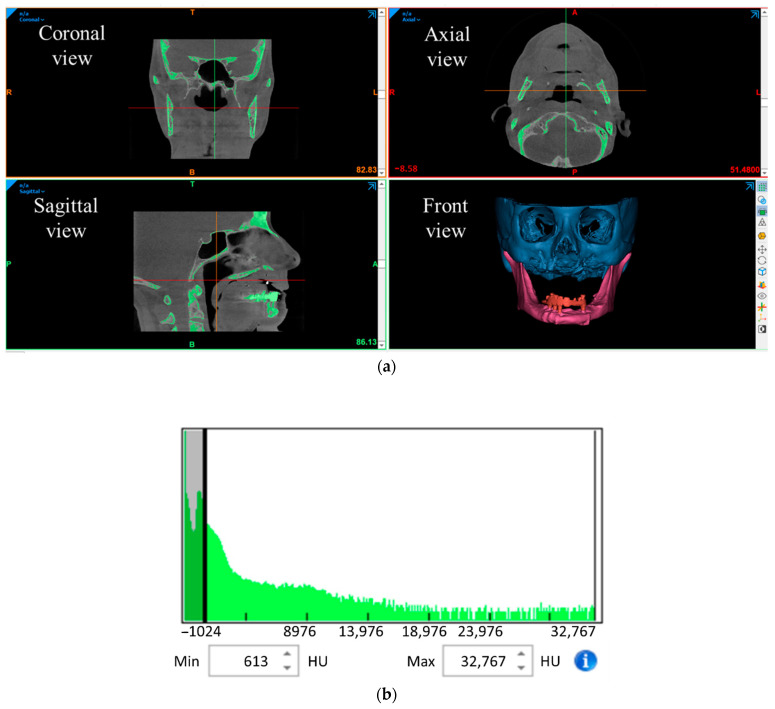
(**a**) Segmentation of maxilla and mandible bones from the overall facial anatomy: coronal, axial, sagittal, and front views; (**b**) Hounsfield radiodensity scale.

A 3D CAD of bone and the dental structure was developed by segmenting the soft tissues out, as shown in Figure 2. The patient’s osteoporotic bone in maxilla structure and absence of maxillary dentition clearly showed the need for maxillofacial and dental implants, respectively. The mandible structure was separated from the maxilla, and a 3D CAD of the maxillary structure was exported in STL format. This data was further used not only in fabricating a biomodel by SLA but also as a reference to develop a patient-specific implant design to ensure a close geometric fit.

#### 2.1.2. 3D-Printed Physical Anatomical Model

A physical model of a biological structure, generally referred to as a ‘biomodel’, has been used in several craniomaxillofacial surgery investigations to not only facilitate and improve treatment planning but to also reduce the risk, time, and cost to patients and hospitals [33,34,35]. The digital biomodel of the patient’s maxilla structure obtained from Mimics in STL format was used to fabricate a physical biomodel using SLA. The STL file was processed through PreForm software (Formlabs, Somerville, MA, USA) that was used for build-setup to define the part layout, orientation, supports, slicing, and printing parameters. An SLA printer, Form 2 (Formlabs, Somerville, MA, USA), was used to build a 3D part through layer-by-layer photopolymerization by ultraviolet (UV) light. After printing, the part was rinsed in isopropyl alcohol (IPA) to remove any uncured resin from its surface. After drying the rinsed part, it was post-cured by exposing it to light and heat to achieve the highest possible strength and stability of the material. Finally, supports were removed from the part and the remaining support marks were sanded for a clean finish.

#### 2.1.3. Implant Design Development

**Figure 3:**
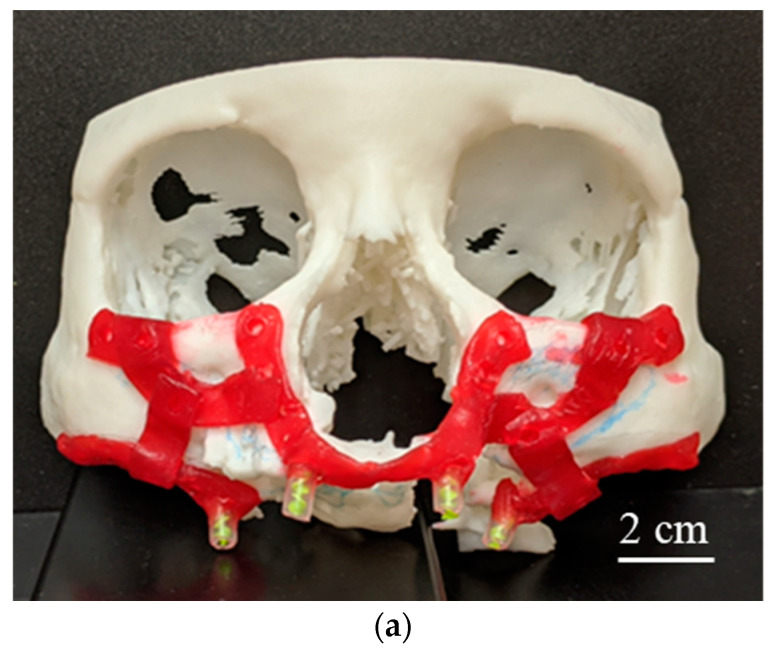

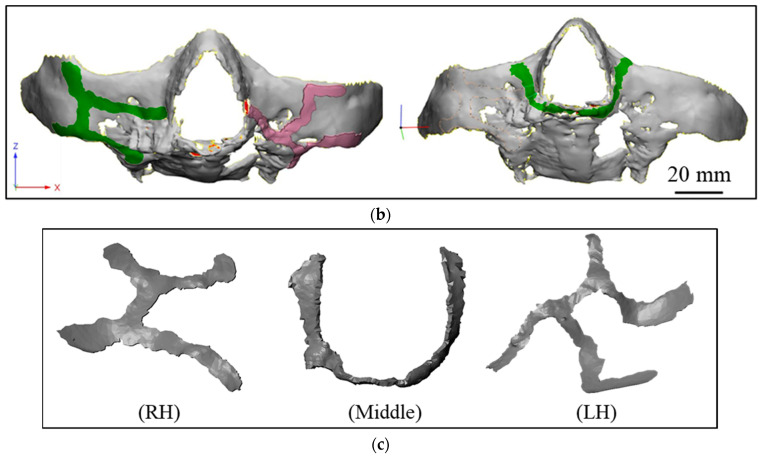
(**a**) Implant design requirement defined by oral/ maxillofacial surgeons considering the current condition of the patient’s maxilla structure bone and dental implant requirements. (**b**) Implant geometries generated from digital biomodel using 3-Matic. (**c**) First-generation design of the implant. The implant was divided into three components, (RH, Middle, and LH parts).

The physical biomodel enhanced visualization and understanding of the current bone loss condition in the patient’s maxilla structure. Figure 3a shows the implant design requirement defined by oral and maxillofacial surgeons after thorough investigations of the patient’s condition, the osteoporotic maxillary bone, and dental implant requirements. In this process, care needed to be taken to ensure the position of important nerves and other soft tissues were investigated while identifying bone with adequate density for fixation of the implant [8]. The maxillofacial implant was split into three components to mitigate surgery difficulties and allow for a certain amount of flexibility in positioning that might be identified during surgery, as indicated by surgeons. Moreover, it was recommended from an engineering point of view as well because of simplification in part design and wider allowable geometric tolerance in fabrication. Moreover, larger support structures would be required to print the implant in one piece, as opposed to smaller supports needed in simplified relatively flatter geometries.

Having developed the design concept, the digital biomodel enabled the development of implant geometry to match the patient’s anatomical condition and identified implant solution. Implant geometries were generated in STL format from digital biomodel using 3-Matic software (Materialise, Leuven, Belgium). Biomodel surfaces were extracted and offset to build the implant geometry as shown in Figure 3b, to ensure a perfect fit between the implant and maxilla structure. For each implant component, an STL file having tessellated surfaces was exported to Solidworks for geometric fine-tuning, edge correction, and STL density reduction. In the proof-of-concept stage, the initial design did not include mounting posts and holes, as the objective was to investigate the applicability of MF^3^ to manufacture such custom implant parts. These models were used for MF^3^ printing of the implant prototypes.

The implant was split into three parts, Right Hand (RH), Middle, and Left Hand (LH) components, as shown in Figure 3c. Here, RH and LH refer to the patient’s LH and RH sides, respectively. Each part consisted of mounting posts in the form of a cylindrical boss that would eventually support dental implants. In addition, mounting holes were provided to mount the implants on the existing maxilla structure of the patient at the best position having sufficient bone density to support the implants.

### 2.2. Fabrication of Customized Ti-6Al-4V Alloy Implant Using MF^3^

An extrusion-based desktop printer, Pulse (MatterHackers, Lake Forest, CA, USA), was used to print the implant prototypes. Green parts were fabricated using 1.75 mm filaments of 59 vol.% of Ti-6Al-4V powder dispersed in a multi-component custom polymer binder. The feedstock and filament were prepared based on our earlier investigations [27,28]. The implant STL file was processed through Simplify3D software to generate GCode instructions. The processing parameters used are shown in Table 1. The printing parameters were selected based on several preliminary printing experiments of various geometries using different parameters. A lower printing speed and smaller layer thickness, as opposed to printing simpler solid geometries, were used to ensure the geometric fidelity of the thin-walled complex geometry of the implants. A layer thickness of 0.1–0.15 mm was chosen to achieve suitable resolution considering the 1 mm thickness of the implant. A 0.4 mm diameter nozzle was selected to achieve a bead width in the range of 0.48–0.60 mm that provides adequate in-plane geometric accuracy. Extrusion and build plate temperatures were chosen in the range of 240–260 °C and 65–75 °C, respectively. A lower printing speed, 5 mm/s, was considered to achieve better detailing of the intricate geometries, as opposed to 10–15 mm/s used generally. A concentric infill toolpath was found more suitable than 0–90° that works well for regular geometries.

#### 2.2.1. Support Structure

**Figure 4:**
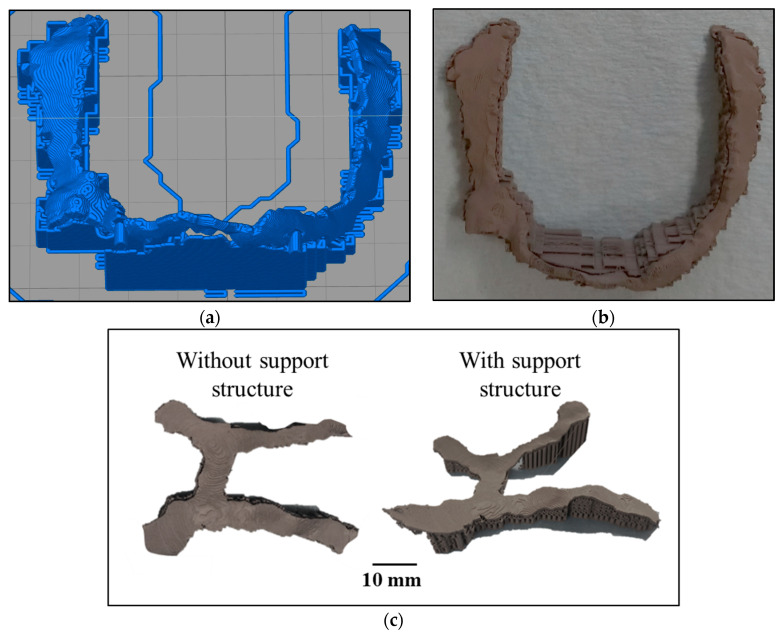
(**a**) Build setup showing sliced model, toolpath support structure of the middle part; (**b**) MF^3^-printed green part; (**c**) printing without appropriate support structure failed and an optimal support led to successful printing of the RH part.

Initial attempts to print the implants by MF^3^ led to poor printability and highly defective parts due to irregular geometry, overhangs, and unsupported features. Hence, the use of a support structure was considered for the first time in MF^3^. Support structure in MF^3^ brings several challenges, such as no sacrificial material can be used for supports that can be dissolved in a solvent because of the risk of losing the integrity of the green part. The other option of support structure using the parent material itself has a challenge that cutting the supports off in the green stage may easily damage the part. Hence, in this study, the support structures printed using the parent material were kept intact through the debinding and sintering stage as well. Moreover, cutting the support off in the sintered metal stage was difficult in this case due to irregular geometry and uneven surfaces of the implant. Hence, minimal support structures were employed to print the thin-walled implants. Eventually, the introduction of support structures improved the printability as shown in Figure 4. For each geometry, an optimal support structure was designed using the slicer tool. All three components were printed, debound, and sintered keeping the support structure that was finally cut off from the sintered part using a diamond-wire machine saw and diamond-wheel handsaw.

#### 2.2.2. Debinding and Sintering

Green parts of the implant components were subsequently subjected to post-printing processes. To completely remove the polymer binder components, a two-step debinding procedure was used to reduce thermal debinding time and debinding-related defects. First, the MF^3^ printed green parts were kept in heptane at 50 °C for 45 min for solvent debinding. After drying, the parts were placed in an oven at 80 °C for 4 h to remove residual solvent. Thermal debinding was carried out in a partial vacuum of 600 mTorr with argon sweep (TM Vacuum Products Inc., NJ, USA at a heating rate of 1 °C/min and held for 3–10 h below 600 °C. Finally, the thermally debound parts were sintered in the same vacuum furnace at temperatures from 1200–1400 °C for 1–4 h with argon as cover gas and a typical heating rate of 3 °C/min [36]. Thermal sintering, finally, provided fully dense Ti-6Al-4V implant prototypes.

#### 2.2.3. Green and Sintered Parts Characterization

The MF^3^ printed green parts were evaluated for the fidelity of geometric profile using an optical surface profiler, Keyence VR 5000 (Keyence, Cincinnati, OH, USA). Moreover, the printed part major dimensions were measured by a Vernier caliper and verified with the original design CAD model dimensions. The sintered parts were characterized for surface integrity including surface topography, density, porosity, microstructure, and hardness that would affect the implant performance. Relative density and porosity were investigated using the Mettler Toledo scale by Archimedes method. In addition, bulk density was calculated that indicates the amount of interconnected open porosity on part surfaces which is not taken into account by Archimedes density. Archimedes density and bulk density together provide an estimation of open interconnected and closed porosities. Surface topography was evaluated by optical microscopy and scanning electron microscopy (SEM) TESCAN VEGA (TESCAN, Brno, Czech Republic). Moreover, surface roughness was measured in terms of average surface roughness parameter, R_a_ along the 0° and 90° to the print direction using a Mitutoyo portable surface roughness tester. The hardness was measured with a Rockwell ‘C’ hardness testing apparatus at 150 Kgf load. For both roughness and hardness, three measurements were taken and the means were calculated. The microstructure was evaluated by etched microscopy and SEM.

### 2.3. MF^3^ Process Simulation

To enable the prediction of MF^3^ printed implant part quality in terms of dimensional variations, warpage, and residual stresses, a thermo-mechanical model was used for finite element simulations using Digimat to simulate the MF^3^ printing process. The 3D CAD model of the implant was imported in STL format in Digimat-AM and discretized into a voxel mesh of element size 0.1 mm. Thermo-mechanical properties of the novel material such as Young’s modulus, specific heat, thermal conductivity, specific volume, and coefficient of thermal expansion were obtained using empirical estimation models from a previously published research work by the authors [27]. Processing parameters used in the simulation were the same as those used in the printing experiments (Table 1). The build plate and ambient temperature define the boundary conditions while the melt extrusion temperature defines the thermal loading. The GCode file from Simplify3D defines the toolpath, layer thickness, and printing speed. Following the toolpath, each layer is activated for the calculation to simulate the physical printing process. Currently, the simulation is not able to consider the presence of or recognize the need for support structures during printing since the software does not include gravity effects in the modeling. This might affect the accuracy of deflection and residual stresses prediction in the areas of unsupported overhangs due to self-weight. The printing process and printed part quality were estimated by post-processing the simulation results. The thermo-mechanical process simulation provided a prediction of part deflection and residual stresses that develop as results of shrinkage and non-uniform cooling that stems from thermal gradient due to layer-by-layer printing. The simulated part dimensions were verified with MF^3^ printed green part dimensions. Moreover, the simulation results can be used in further optimization of the implant design, in future studies.

## 3. Results

### 3.1. MF^3^ Printed Green Parts

**Figure 5:**
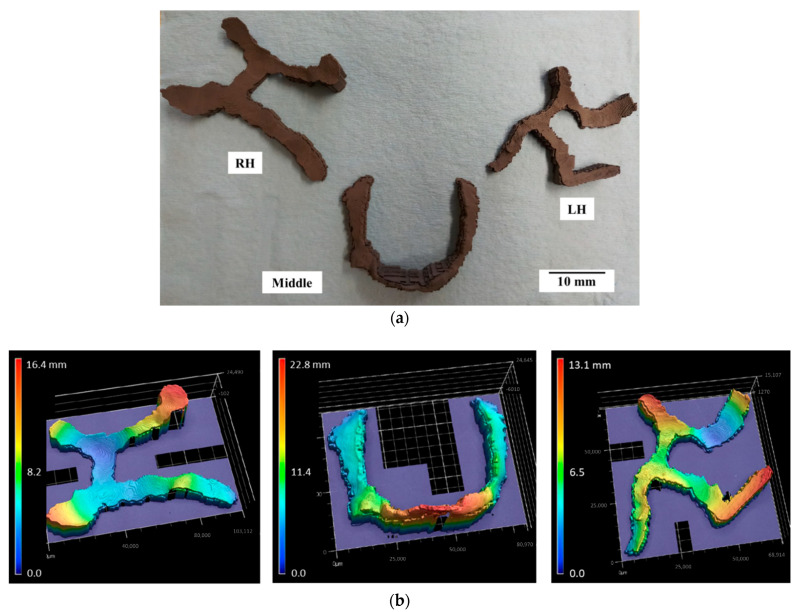
(**a**) Green parts with a support structure; (**b**) optical surface profilometry of maxillofacial implants.

Figure 5a shows the printed green parts of all three components (RH, Middle, and LH) of the maxillofacial implant. The support structure of each part was generated by the slicing software depending on part geometry and orientation on the print bed. Post printing, this support was further kept intact to retain part geometry and minimize potential damage in the green stage. Moreover, the removal of the support structure at this stage had associated risks of part damage. Hence, support structures were not removed in the green stage. The geometric fidelity of printed parts was evaluated using an optical surface profiler, as shown in Figure 5b. It enabled the verification of maxillofacial implants with complex unique geometries that cannot be measured using conventional scales. The Z-axis positioning of millions of scanned points on the surface is plotted that can be used to verify the accuracy with original 3D CAD geometry. The surface profile generated by the tool can further be processed through a CAD tool to develop a surface geometry CAD model and overlapped with the original geometry STL model to verify the deviations in shape and size. Moreover, this data is useful in surface roughness investigations.

### 3.2. Printing Process Simulations

**Figure 6:**
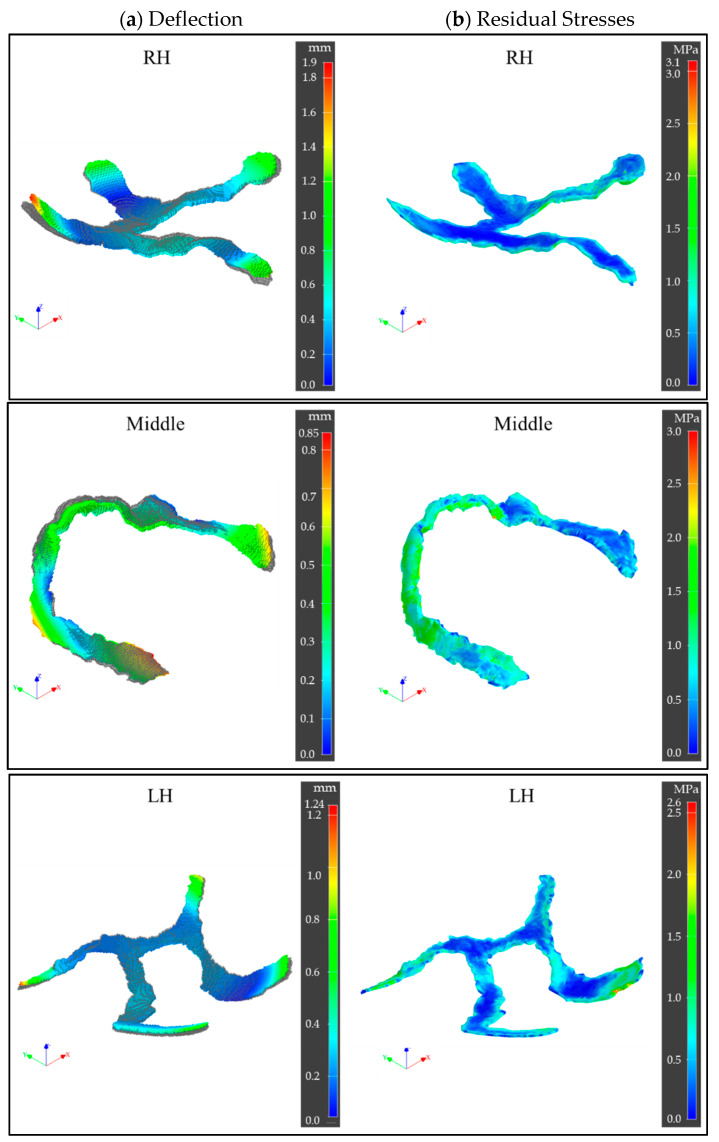
MF^3^ printing process simulation results. (**a**) Part deflection overlapped on original CAD design; (**b**) residual stresses (von Mises) estimation, in RH, Middle, and LH parts.

In the MF^3^ printing process simulations, the sequential thermo-mechanical analyses by Digimat provided an estimation of deflections in X, Y, Z directions as well as the overall deflection, and residual stresses in the printed part. The layer-by-layer printing process led to thermal strain that eventually caused residual stresses, deflection, and warpage, finally leading to deviations in printed part dimensions and shape as opposed to the original CAD geometry as shown in Figure 6.

The implant edges were found to experience a higher rate of heat loss by convection and faster cooling due to larger surface areas, leading to earlier crystallization and solidification than the central regions. The resulting non-uniform volumetric shrinkage caused greater defection and residual stresses at these locations. Moreover, the lack of structural constraints at these free ends contributed to large deflections. Maximum deflection in the RH, Middle, and LH implant ends were observed to be 1.9 mm, 0.85 mm, and 1.24 mm, respectively, while in the central zone the deflections were as low as zero. The difference among the parts can be attributed to geometry aspect ratio (length/thickness), structural stiffness, and overhang length difference. The LH part with a relatively higher aspect ratio, lower structural stiffness, and larger overhang length led to higher deflections while the Middle part showed the least. Figure 6 also indicates the von Mises stress as residual stresses developed at the end of printing. Maximum residual stresses in the RH, Middle, and LH parts were observed to be 3.1 MPa, 3 MPa, and 2.6 MPa, respectively. Differential heat transfer and thermal gradient along the print direction (Z-axis) as well as across print cross-section (XY-plane) led to such differences among parts and within a single part.

**Figure 7:**
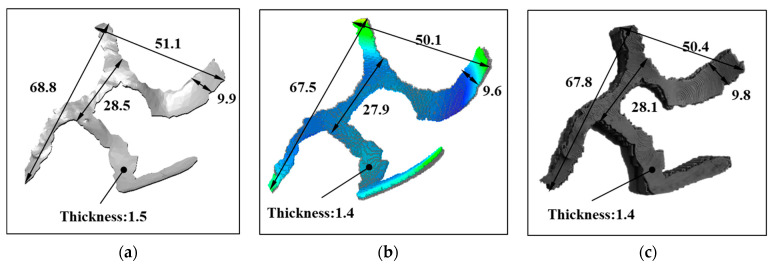
Typical dimensions of LH part of the customized implant. (**a**) CAD design; (**b**) simulation estimation; (**c**) printed green part.

For quantitative verification of simulation results, simulated part dimensions were measured and compared with experimental results. Figure 7 shows the dimension results from simulation and experiments compared with the original CAD dimensions of the LH part. Experimental part dimensions fairly matched with that of the simulated part. Simulated parts showed an overall shrinkage of 1.96% from CAD dimensions, whereas in experiments it was found to be 1.37%. Including support structure in simulations, the accuracy of estimation could further be improved. Moreover, while the same endpoints dimensions in the CAD model, simulated part, and physical part were measured, the dimension differences could be attributed to slight differences in the measurement locations.

### 3.3. Sintered Ti-6Al-4V Parts Characterization

**Figure 8:**
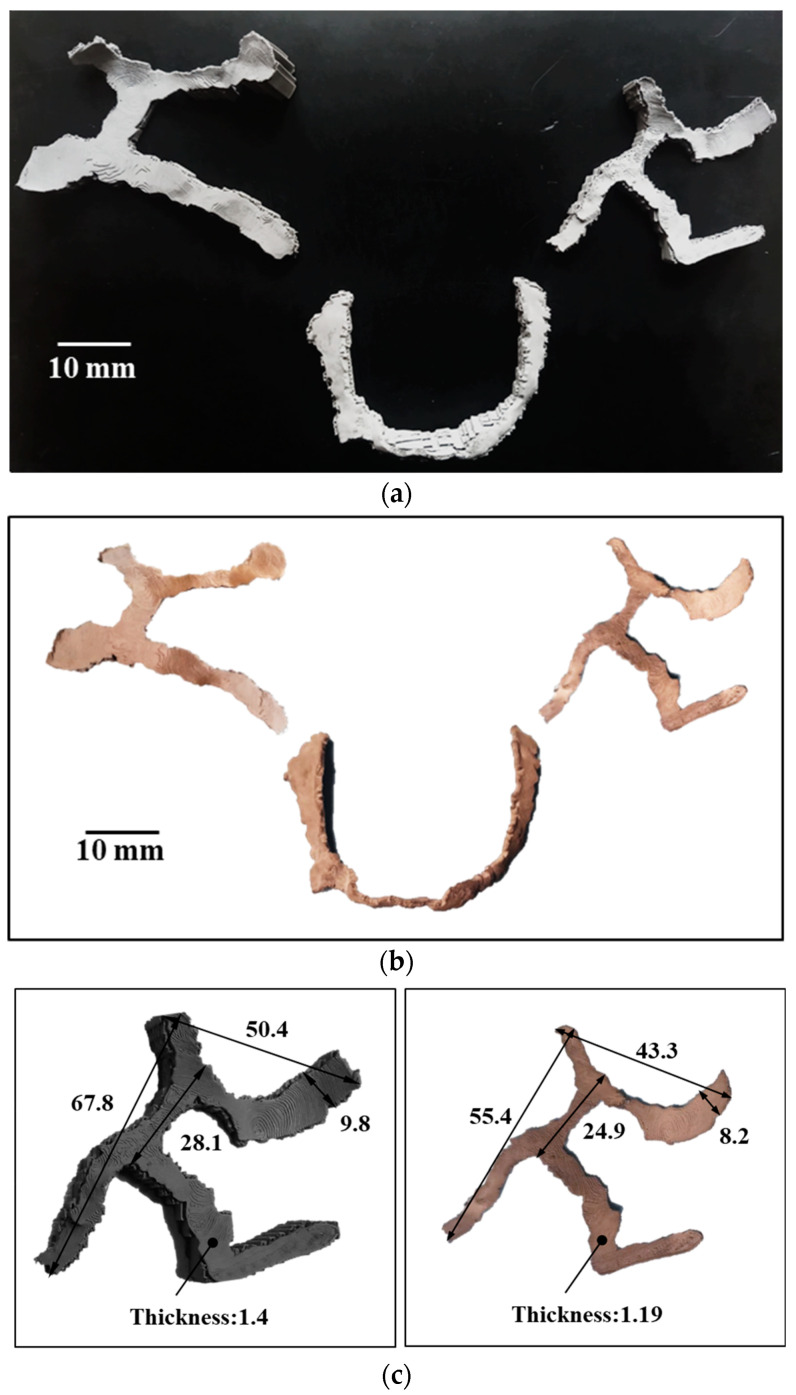
(**a**) Sintered metal parts with a support structure; (**b**) sintered metal parts after support structure removal; (**c**) green part vs sintered part dimensions showed 16% shrinkage in sintering.

Support structures were retained during debinding and sintering processes to avoid a potential collapse of unsupported geometry and minimize part distortion. Figure 8a shows the sintered Ti-6Al-4V implant components with the support structure. Diamond wire machine saw and diamond wheel hand saw were used to gradually cut the supports off. Figure 8b shows the sintered implant components without a support structure.

#### 3.3.1. Surface Topography

**Figure 9:**
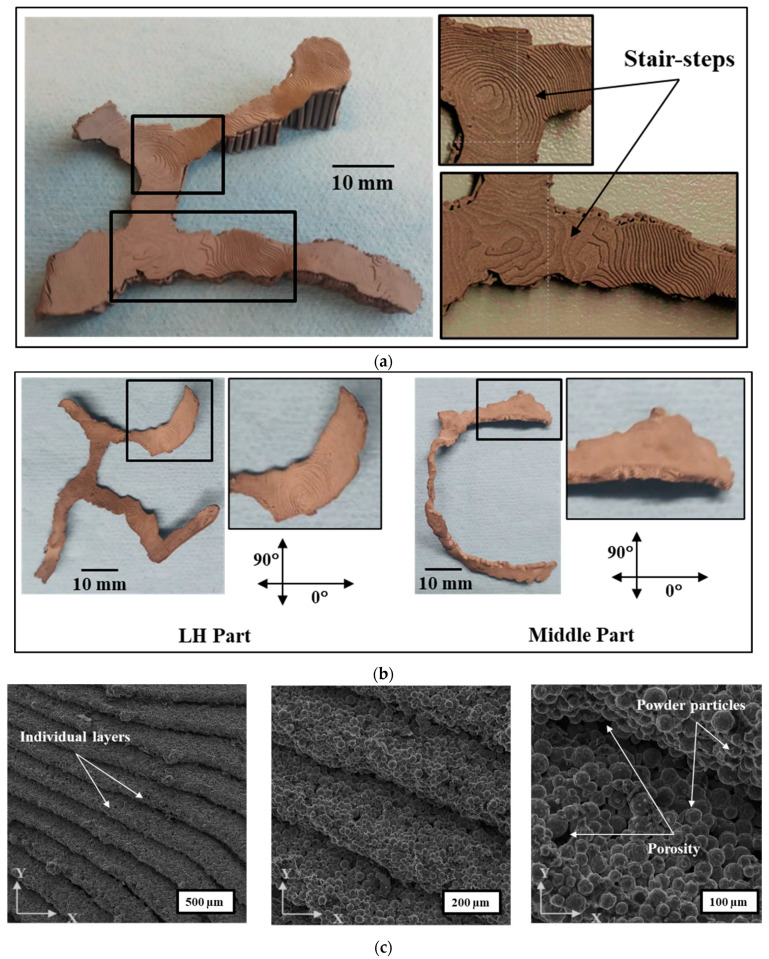
(**a**) Stair-step effects from layer-by-layer printing due to Z-gradient of the implant surface; (**b**) surface roughness of LH and Middle unpolished sintered parts measured in terms of ‘R_a_’ along the 0° and 90° of the print direction; (**c**) SEM (unetched unpolished condition).

A considerable stair-steps effect was observed in the sintered implant parts as shown in Figure 9a. Hence, a finer layer thickness was chosen to minimize this effect and get higher exactness to the CAD geometry. Moreover, the layer-by-layer and bead-by-bead printing by the extrusion-based process of MF^3^ printing leads to surface roughness that follows the toolpath as shown in high magnification of SEM micrographs in Figure 9c. This could be attributed to the lack of diffusion between layers and beads.

Moreover, the overall surface roughness caused by the combined effects of stair-step and lack of layer-to-layer and bead-to-bead diffusion depends on part orientation and surface angle with the horizontal plane. Figure 9b shows the surface roughness measured on as-sintered parts along 0° and 90° of the print direction. The difference in part geometry and orientation on the print bed led to different surface angles and toolpath, hence, the variation in surface roughness, accordingly, as shown in Table 2. The average surface roughness parameter, Ra, was used that provides the arithmetic average of surface heights measured across a surface. LH part showed higher roughness (Ra: 23.3 µm) in 0° than that of the Middle part (Ra: 13.5 µm), while in 90°, both parts showed the same results (Ra: ~12.7 µm). Higher surface roughness was observed with a higher surface angle with the build plate (horizontal plane). Hence, part orientation in the build plate becomes an important aspect apart from other slicing and printing parameters such as layer thickness, bead width, and extrusion temperature that affect surface roughness.

The SEM images in Figure 9c further show the implant surface topography on different scales. The stair-step effect at layer thickness level shows a typical pattern that stems from part geometry, part orientation, and slicing strategy (toolpath, layer thickness, bead width). This stair-step contributes to macro-level surface roughness. Secondly, at the individual layer level, powder particles and porosity can be seen that contribute to micro-level surface roughness. As the surface topology is extremely important for an implantable medical device and it has to be controlled [37,38]. Hence, further investigation and optimization of surface roughness would be worth looking into this aspect.

#### 3.3.2. Relative Density and Porosity

The printed samples were characterized for density using the Archimedes method, refer Table 3. Sintered metal parts were evaluated for relative density and porosity considering Ti-6Al-4V has a theoretical density of 4.23 g/cc. The relative density (bulk density-based) of the Middle part was found to be 81% indicating the total porosity (containing both open interconnected porosity and closed porosity) of 19%. Archimedes-based relative density was 94% indicating 6% closed porosity, hence, 13% open interconnected porosity. These results indicate a considerable amount of interconnected open porosity.

**Figure 10:**
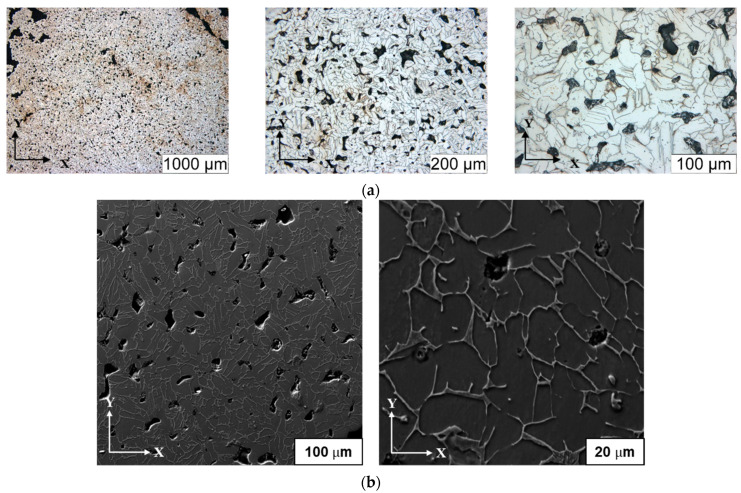
(**a**) Optical microscopy (etched polished condition); (**b**) SEM (etched polished condition).

The optical micrographs revealed considerable porosities with sizes of 50 µm as shown in Figure 10a. A porosity of 750 µm seems to be the best for cell infiltration while smaller pores provide higher mechanical properties [39]. Using 3D printing, the size of the pores can be adapted to a specific purpose.

#### 3.3.3. Metallography

The grains were equiaxed showing no preferred orientation for their coarsening. The grain size was measured to be 15 ± 2 µm. Further characterization was performed using an optical microscope to analyze the existing phases on the etched microstructure as shown in Figure 10. Using a Rockwell hardness tester, the hardness of the printed implant samples was measured, and 6.52 ± 0.8 HRC was observed. For EBM and SLM printed parts, it was found to be 37–57 HRC [40].

## 4. Discussions

Having followed through the specific digital workflow and using suitable support structures, all three components (RH, Middle, and LH) of the maxillofacial implant prototypes were successfully printed by MF^3^ of Ti-6Al-4V. Furthermore, MF^3^ printing process simulations were conducted by modeling the layer-by-layer printing of the extrusion-based process. The thermal gradient combined with non-uniform cooling during printing led to inherent thermal strains in the printed parts. The strain varies according to the thermal gradient and coefficient of thermal expansion (CTE) of material [41]. The inherent strain further leads to residual stresses that subsequently distort the printed part and also affect its mechanical strength. In MF^3^, high residual stresses may also lead to cracks or damage the part during the debinding and sintering processes. Lower thermal gradient, uniform, and slower cooling would help in reducing residual stresses. While the simulation of the MF^3^ printing process enabled a fair estimation of printed green part geometry and residual stresses, it is important to note that currently, the simulation tool does not consider support structure in modeling. It may affect the accuracy of simulation prediction.

During debinding and sintering processes, while the support structure helped in retaining the shape and minimizing distortion in the thin-walled implant parts, on the other hand, it was extremely challenging to cut the support off implant geometry in a fully sintered metal phase. Firstly, cutting the supports off the sintered parts was not easy for conventional metal cutting methods, particularly, due to the irregular geometry and wavy surfaces of the implants. Secondly, thin-walled geometry was part of the problem because the parts could easily be damaged while chipping the supports off. Moreover, the vertical walls of the support structure were thicker than the part itself, contributing to the possibility of part breakage. These issues can be addressed by investigating the feasibility of maximum angle and length of unsupported overhang that can be printed by MF^3^. In addition, the design and optimization of the support structure are needed to achieve adequate support using a minimal structure. However, these aspects were beyond the scope of the current investigation.

In MF^3^ printing, as a 3D model is discretized into horizontal layers, the presence of a sharp change in the curvature of the implant surface causes stair-steps effect. Hence, the maxillofacial implant complex surface profile matching human anatomy developed significant stair-step effects. The offset between adjacent layers having varying cross-sections along the print axis led to such deviations from the desired geometry. It further contributed to the surface roughness of the implants. Moreover, a considerable amount of interconnected open porosity was observed in the sintered parts. The open interconnected porosity has engaging characteristics that accelerate the healing of the bone and enhance the osteointegration of metallic implants [3]. Such porosity provides anchor sites to the bone tissue and promotes accelerated osseointegration. By optimizing the open interconnected pore system, osseointegration can be biologically enhanced in implants. Moreover, microporosity better mimics the natural bone in terms of elastic modulus (cancellous: 1.5–11.2 GPa and cortical: 7–20 GPa) as opposed to fully dense Ti-6Al-4V (105 ± 2 GPa) [42,43]. This, in turn, leads to a more uniform stress distribution between the implant and adjoining bones. The lower hardness value of MF^3^ printed implants could be attributed to the porosity that can be further investigated and optimized. However, the lower hardness value of MF^3^ printed implants as opposed to EBM and SLM printed parts would mimic the bone characteristics more effectively and favor the implant performance as it could better match the bone hardness which is 40–44 HV [44].

### Future Work

The findings from this study will further allow the development of a beta version of the implants that would enable the dental research team to test and validate through surgical procedures on patient-specific biomodels. It would include refined geometries having smooth curves and surfaces developed matching the patient’s anatomy. In addition, multiple mounting posts for dental implants and holes for mounting the implants on the maxilla structure are to be provided. In future work, the second-generation design is to be fabricated by MF^3^ and the beta prototype tested for the clinical procedure.

Moreover, to achieve the highest performance, suitable heat treatment requirements are to be investigated to relieve residual stresses, improve ductility and toughness, and attain desired microstructures. Following the sintering process, the implant may be subjected to hot isostatic pressing, surface nitriding, polishing, and chemical cleaning to enhance biological performance. In addition, corrosion of the material can severely limit its fatigue life and mechanical strength. Even though titanium alloys are exceptionally corrosion-resistant because of the stability of the TiO_2_ oxide layer, they are not inert to corrosive attack. Hence, maxillofacial implants need to be tested for corrosion in solutions that mimic biofluids like blood.

## 5. Conclusions

MF^3^ printing of custom maxillofacial implant prototypes was studied for the first time using experimental and analytical investigations. A methodology for fabrication of patient’s biomodel and custom maxillofacial implants using additive manufacturing technology is demonstrated. The sintered metal implant prototypes were characterized for surface integrity properties such as density, porosity, surface roughness, hardness, and microstructure that play important role in the performance of an implant.

The following conclusions emerge from the study:

1. Fabrication of patient-specific custom maxillofacial implant prototypes out of Ti-6Al-4V by MF^3^ has been found feasible and demonstrated through the experimental study. However, as the design was in a preliminary stage, some critical parts such as holes and mounting posts were not included in this study. Further investigations are still needed to investigate fully equipped prototypes for a real assessment and final validation.
2. A specific digital workflow is required to convert the patient’s CBCT data into a 3D printable format that made additive manufacturing of the anatomical model and the maxillofacial implant prototypes possible.
3. MF^3^ printing with support structures was reported for the first time. Optimal support structures were required in MF^3^ for custom implant geometries to ensure geometric fidelity not only during printing but also debinding and sintering processes.
4. MF^3^ process simulation estimated maximum deflections of 0.9–1.9 mm and maximum residual stresses of ~3 MPa in printed green parts. However, the accuracy of prediction would be affected by the absence of support structures in simulations as opposed to experimental printing.
5. The relative density (bulk density-based) of the Middle part was found to be 81% indicating the total porosity of 19% including 6% closed porosity and 13% open interconnected porosity that would provide anchor sites to the bone tissue and promotes accelerated osseointegration.
6. Stair-step effects and lack of diffusion between layers contributed to surface roughness at the macro scale, whereas powder particles and porosity within a layer resulted at the micro-scale. LH part showed higher roughness (Ra: ~23 µm) in 0° than that of Middle part (Ra: 13 µm), while in 90° both parts showed the same results (Ra: ~13 µm). The difference in part geometry and orientation on the print bed led to different surface angles and toolpath, hence the variation in surface roughness, accordingly. Higher surface roughness was observed with a higher surface angle with the build plate.
7. The hardness of 6.5 ± 0.8 HRC was observed in the Ti-6Al-4V implants printed by MF^3^ as opposed to 45 ± 10 HRC in EBM and SLM.

The outcome of the work proves that MF^3^ is a potential process to manufacture patient-specific custom implants out of Ti-6Al-4V. It also represents a part of the treatment procedure for complex surgery in elderly patients with a severely atrophic posterior maxilla eliminating the need for regenerative bone therapies. Moreover, it was demonstrated how additive manufacturing technologies could help the surgeon to improve pre-operative planning in implant surgery.

## Figures and Tables

**Table 1:** Printing process parameters.

Process Parameters	Layer Thickness (mm)	Bead Width (mm)	Extrusion Temperature (°C)	Build Plate Temperature (°C)	Extrusion Multiplier	Printing Speed (mm/s)	Toolpath
Settings	0.1	0.48	240	65	1	5	Concentric

**Table 2:** Surface roughness of as-sintered parts.

Part	Measurement Angle	Ra (µm)
LH	0°	23.3 ± 1.0
	90°	12.9 ± 1.2
Middle	0°	13.5 ± 1.0
	90°	12.7 ± 0.7

**Table 3:** Relative density and porosity.

	Archimedes Density (g/cc)	Relative Density (%) (AD-Based)	Bulk Density (g/cc)	Relative Density (%) (BD-Based)
Middle part	4.18	94.3	3.60	81.2
